# Chondroitinase ABC reduces dopaminergic nigral cell death and striatal terminal loss in a 6-hydroxydopamine partial lesion mouse model of Parkinson’s disease

**DOI:** 10.1186/s12868-019-0543-3

**Published:** 2019-12-20

**Authors:** Edward J. R. Fletcher, Lawrence D. F. Moon, Susan Duty

**Affiliations:** King’s College London, Institute of Psychiatry, Psychology and Neuroscience, Wolfson Centre for Age-Related Diseases, Guy’s Campus, London, SE1 1UL UK

**Keywords:** Parkinson’s disease, Chondroitinase ABC, 6-Hydroxydopamine, Neuroprotection, Chondroitin sulphate proteoglycans

## Abstract

**Background:**

Parkinson’s disease (PD) is characterised by dopaminergic cell loss within the substantia nigra pars compacta (SNc) that leads to reduced striatal dopamine content and resulting motor deficits. Identifying new strategies to protect these cells from degeneration and retain striatal dopaminergic innervation is therefore of great importance. Chondroitin sulphate proteoglycans (CSPGs) are recognised contributors to the inhibitory extracellular milieu known to hinder tissue recovery following CNS damage. Digestion of these molecules by the bacterial lyase chondroitinase ABC (ChABC) has been shown to promote functional recovery in animal models of neurological injury. Although ChABC has been shown to promote sprouting of dopaminergic axons following transection of the nigrostriatal pathway, its ability to protect against nigrostriatal degeneration in a toxin-based module with better construct validity for PD has yet to be explored. Here we examined the neuroprotective efficacy of ChABC treatment in the full and partial 6-hydroxydopamine (6-OHDA) lesion mouse models of PD.

**Results:**

In mice bearing a full 6-OHDA lesion, ChABC treatment failed to protect against the loss of either nigral cells or striatal terminals. In contrast, in mice bearing a partial 6-OHDA lesion, ChABC treatment significantly protected cells of the rostral SNc, which remained at more than double the numbers seen in vehicle-treated animals. In the partial lesion model, ChABC treatment also significantly preserved dopaminergic fibres of the rostral dorsal striatum which increased from 15.3 ± 3.5% of the intact hemisphere in saline-treated animals to 36.3 ± 6.5% in the ChABC-treated group. These protective effects of ChABC treatment were not accompanied by improvements in either the cylinder or amphetamine-induced rotations tests of motor function.

**Conclusions:**

ChABC treatment provided significant protection against a partial 6-OHDA lesion of the nigrostriatal tract although the degree of protection was not sufficient to improve motor outcomes. These results support further investigations into the benefits of ChABC treatment for providing neuroprotection in PD.

## Background

Parkinson’s disease (PD) is typically characterised by a range of cardinal motor symptoms that result from dopaminergic cell loss within the substantia nigra pars compacta (SNc) [1]. To date, no treatment has been successful in preventing or reversing this cell loss and the search for new therapies continues. The remaining SNc cells possess limited capabilities for axonal repair and regeneration, a trait that is likely caused by the growth inhibitory environment found within the adult CNS. Major contributors to this inhibitory environment are the chondroitin sulphate proteoglycans (CSPGs) of the extracellular matrix. Interestingly, CSPGs are found as inclusions within neurones and amongst astrocytes in human PD brains at post-mortem [2]. CSPGs have been extensively researched as potential targets for repair in CNS injuries [3], most notably spinal cord injury. Researchers have used the enzyme chondroitinase ABC (ChABC) to digest the CSPG’s glycosaminoglycan sidechains (CS-GAGs) and promote neuroplasticity and repair [4–6]. Digestion of CS-GAGs by ChABC is also proposed to liberate bound molecules such as trophic factors [7] that may provide a more pro-survival environment for both uninjured and degenerating neurones, thereby providing potential neuroprotective efficacy.

To date, the application of ChABC in the PD field has centred on demonstrating the ability of CS-GAG digestion to improve the success of dopaminergic cell replacement therapies [8–11], rather than on its ability to provide protection or repair of endogenous dopaminergic SNc cells. However, Moon et al. [12] administered ChABC to the rat nigrostriatal tract following Scouten knife axotomy and observed significant dopaminergic fibre sprouting back to the striatum. Subsequent studies identified similar efficacy of ChABC in nigrostriatal Scouten Knife models [13–15] suggesting that ChABC is capable of promoting repair in the rodent nigrostriatal pathway just as within the spinal cord. However, as this axotomy model does not replicate the pathology associated with PD, whether ChABC may help reduce SNc cell and fibre loss via a protective mechanism in a parkinsonian brain remains uncertain.

Here we examined the disease modifying potential of ChABC in the unilateral 6-hydroxydopamine (6-OHDA)-lesioned mouse which has good construct validity, replicating the mitochondrial dysfunction, oxidative stress and neuroinflammatory aspects of PD [16, 17]. Accordingly, in this model, the microenvironment in which the CSPGs exist contains not only damaged or degenerating SNc cells, but also a range of reactive oxygen species, reactive astrocytes and microglia [17]. We hypothesised that ChABC will elicit neuroplasticity and protection within the 6-OHDA injured nigrostriatal pathway leading to improved motor outcomes. To test this, we measured both behavioural outcomes and the extent of SNc cell and fibre loss in this model following ChABC administration. Furthermore, as PD is a progressive disease we set out to determine the efficacy of ChABC treatment in both late- and early-stage PD, replicated here by inducing full (> 90% SNc cell loss) and partial (50–60% SNc cell loss) 6-OHDA lesions, respectively [18–20].

## Results

### Chondroitinase ABC treatment does not reduce nigrostriatal pathology or motor impairment in mice bearing a unilateral full 6-OHDA lesion of the nigrostriatal tract

**Fig. 1:**
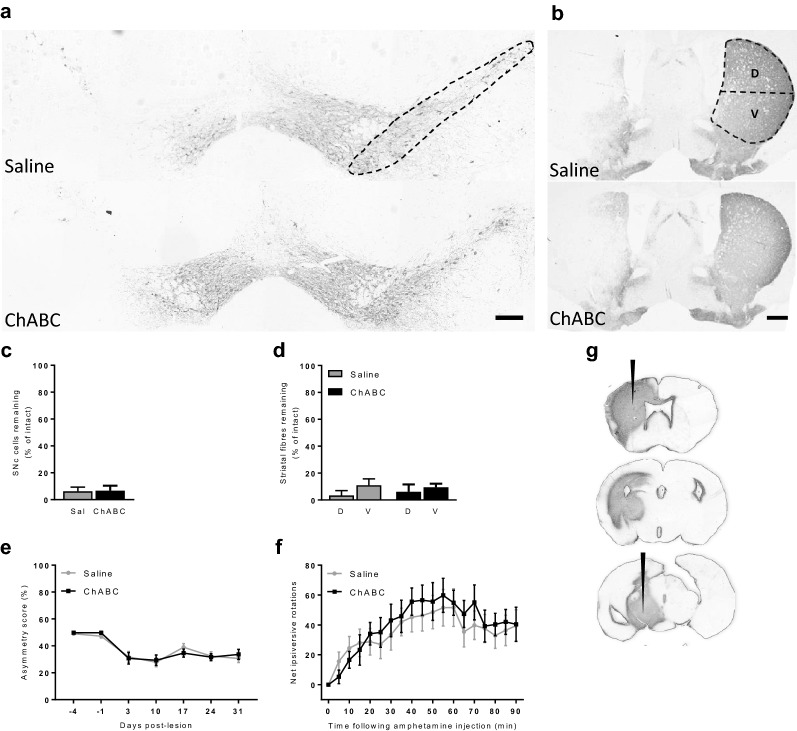
ChABC fails to improve cellular or motor outcomes in the full 6-OHDA lesion mouse model. Representative TH-stained coronal sections of **a** rostral SNc and **b** striatum from animals that received saline (top panel) or ChABC (bottom panel) into the 6-OHDA lesioned hemisphere (left hand side). Regions analysed for SNc cell counts and TH-fibre mean grey value (MGV) are outlined. **c** The number of SNc cells remaining and **d** TH-positive fibre MGV within the dorsal (D) and ventral (V) striatum are quantified in the lesioned hemisphere as a percentage of the intact hemisphere. Data are averaged across all three rostrocaudal levels. No significance was detected between saline- and ChABC-treated animals. Similarly, no difference was observed between saline- or ChABC-treated animals in either **e** asymmetry score or **f** net amphetamine induced ipsiversive rotations. Saline (Sal): n = 14 and ChABC: n = 13. Data are mean ± SEM. Scale bars: a = 200 µm; b = 1000 µm. **g** ChABC-mediated digestion was confirmed by C4S immunoreactivity. Digestion of the CSPGs, evidenced by C4S stain, was detected along the entire nigrostriatal tract from the SNc level (bottom section) to the striatal level (top section). Black arrows indicate ChABC injection sites

Absolute numbers of SNc cells per section for the intact hemisphere were consistent with previously published SNc cell count data [21, 22]: full lesion vehicle group, 92 ± 4.4; full lesion ChABC group, 86 ± 2.4. ChABC digestion of CS-GAGs along the nigrostriatal tract failed to provide any protection to either SNc cells or TH-positive fibres in the unilateral full 6-OHDA lesion mouse model. Animals that received ChABC showed no significant difference in the percentage of cells remaining in their lesioned hemisphere (6.7 ± 3.7% of intact) compared to those that had received saline (6.2 ± 3.1% of intact; *p* = 0.46, *t* = 0.09, *df* = 25; Fig. 1a, c). Furthermore, TH-positive fibre density within the dorsal or ventral striatum in the ChABC group (6.1 ± 5.4% and 9.5 ± 2.7%, respectively) was not significantly different from that in the dorsal or ventral striatum of control animals (3.4 ± 3.5% and 10.9 ± 4.8%, respectively; two-way ANOVA; *F*_1,50_ = 0.01, *p* = 0.89; Fig. 1b, d). Following 6-OHDA lesioning, there was a significant decline in cylinder test asymmetry scores, reflecting reduced use of the contralateral forepaw, in both groups as an effect of time (two-way repeated measures ANOVA; *F*_6,150_ = 20.1, *p* < 0.001; Fig. 1e). However, there was no significant effect of ChABC treatment on asymmetry score (two-way repeated measures ANOVA; *F*_1,25_ = 0.01, *p* = 0.91). Similarly, there was no effect of treatment, only time, on the degree of amphetamine-induced net ipsiversive rotations produced over the 90 min recording period (two-way repeated measures ANOVA; *F*_1,25_= 0.17, *p* = 0.68; Fig. 1f).

To confirm that ChABC had digested CSPGs within the nigrostriatal tract, immunohistochemistry was used to stain for the chondroitin-4-sulphate (C4S) stub antigen that remains following ChABC-mediated digestion. C4S-immunoreactivity was detected along the entire nigrostriatal tract, including the SNc and striatum, of ChABC-treated animals (Fig. 1g). Subsequent incubation with ChABC ex vivo did not further increase C4S immunoreactivity confirming that full digestion of CSPGs had already been achieved in vivo. Saline-treated animals presented no C4S-immunoreactivity confirming no CSPG digestion in these control animals (images not shown).

### Chondroitinase ABC reduces nigrostriatal pathology without improving motor function in mice bearing a unilateral, partial 6-OHDA lesion of the nigrostriatal tract

**Fig. 2:**
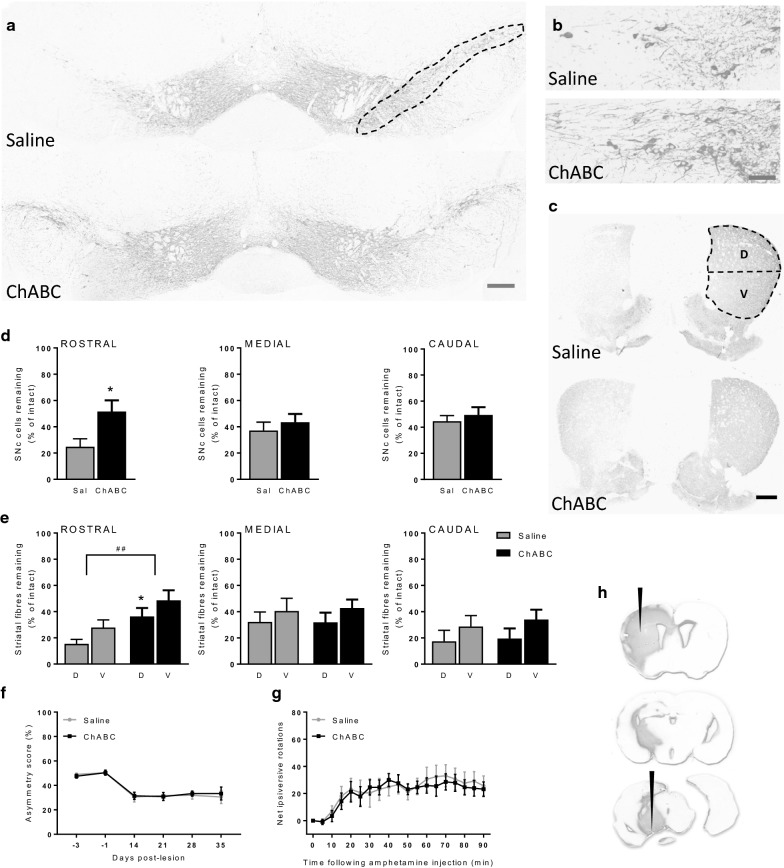
ChABC improves cellular but not behavioural outcomes in the partial 6-OHDA lesion mouse model. Representative TH-stained coronal sections of **a**, **b** rostral SNc and **c** striatum from animals that received saline (top panel) or ChABC (bottom panel) into the 6-OHDA lesioned hemisphere (left hand side). Animals treated with ChABC show enhanced preservation of SNc cells and TH-positive fibres. Regions analysed for cell counts and TH-fibre mean grey value (MGV) are outlined. **d** Quantification of the number of SNc cells remaining in the lesioned hemisphere as a percentage of the intact hemisphere. ChABC treatment significantly protected cells in the rostral SNc when compared to control (*p = 0.02; unpaired t-test) but showed no protection at medial or caudal levels. **e** Quantification of the TH-positive fibre MGV within the lesioned striatum as a percentage of the intact striatum. While no differences were noted within either the medial or caudal striatum, the rostral striatum of ChABC-treated animals showed a group-wise increase in TH-positive fibre MGV (^##^p = 0.002). Post-hoc analysis revealed TH-positive fibre density was significantly preserved in the dorsal (D) rather than ventral (V) aspect of the ChABC-treated rostral striatum when compared to controls (*p = 0.049; Bonferroni post-hoc). No change was detected between saline- or ChABC-treated animals in either **f** asymmetry score or **g** net amphetamine-induced ipsiversive rotations. Saline (Sal): n = 17 and ChABC: n = 17. Data are mean ± SEM. Scale bars: **a** = 200 µm; **b** = 50 µm; **c** = 1000 µm. **h** ChABC-mediated digestion was confirmed by C4S immunoreactivity. Digestion of the CSPGs, evidenced by C4S stain was detected along the entire nigrostriatal tract from the SNc level (bottom section) to the striatal level (top section). Black arrows indicate ChABC injection sites

ChABC treatment provided significant preservation of both SNc cells and striatal TH-positive fibres in the unilateral partial 6-OHDA lesion mouse model. Within the SNc, a significant difference in the percentage of cells remaining in the lesioned hemisphere was found between the saline- (24.8 ± 6.1% of intact hemisphere) and ChABC-treated animals (51.6 ± 8.5% of intact hemisphere) at the rostral level (*p* = 0.02, *t* = 2.4, *df* = 32; Fig. 2a, d) but not at either the medial (*p* = 0.48, *t* = 0.68, *df* = 32; Fig. 2d) or the caudal (*p* = 0.53, *t* = 0.6, *df* = 32; Fig. 2d) levels. Further visual inspection of the rostral SNc revealed an apparent increase in density of neurites in ChABC-treated versus saline-treated animals (Fig. 2b). This was reflected in a significant increase in overall TH density in the rostral SNc in ChABC-treated animals (74.8 ± 9.2% of intact hemisphere) compared to saline-treated animals (39.6 ± 7.8% of intact hemisphere) (*p* = 0.007, *t* = 2.9, *df* = 32).

Within the striatum, TH-positive fibres were also significantly preserved at the rostral level following ChABC treatment (two-way ANOVA; *F*_1,64_ = 10.6, *p* = 0.002). Post-hoc analysis revealed a significant effect in dorsal striatum alone (p = 0.049; Fig. 2c, e) where saline control animals retained 15.3 ± 3.5% TH-positive fibres whereas ChABC-treated animals retained 36.3 ± 6.5%. As with the SNc, there was no effect of ChABC treatment in either the medial (two-way ANOVA; *F*_1,64_= 0.01, *p* = 0.9; Fig. 2e) or caudal (two-way ANOVA; *F*_1,64_ = 0.2, *p* = 0.64; Fig. 2e) levels of the striatum.

As per the full lesion study, animals bearing a partial 6-OHDA lesion showed a significant reduction in asymmetry score as a result of time (two-way repeated measures ANOVA; *F*_5,160_ = 26, *p* < 0.001; Fig. 2f). However, there was no significant effect of treatment (two-way repeated measures ANOVA; *F*_1,32_ = 2.45, *p* = 0.129; Fig. 2f). Furthermore, there was no effect of treatment on the degree of amphetamine-induced net ipsiversive rotations (two-way repeated measures ANOVA; *F*_*1,32*_ = 0.05, *p* = 0.82; Fig. 2g).

C4S stub immunoreactivity was again evident throughout the extent of the nigrostriatal tract in ChABC-treated animals, confirming effective CSPG digestion (Fig. 2h). Conversely, saline-treated animals presented no C4S- immunoreactivity confirming no CSPG digestion in these control animals (images not shown). Additionally, as with the full lesion study, ChABC ex vivo incubation did not further increase C4S immunoreactivity in the partially lesioned tissue confirming the full digestion of CSPGs had been achieved in vivo.

## Discussion

Although treatment with ChABC has previously been shown to aid repair of the nigrostriatal tract following transection [12–15], no study to date has explored the efficacy of ChABC as a neuroprotective agent in a toxin-based model of PD that better reflects the neurodegenerative nature of the human condition. This study therefore set out to examine whether ChABC-mediated digestion of CS-GAGs could protect the nigrostriatal tract against toxic insult using the well-characterised 6-OHDA lesion model of PD that harnesses oxidative stress, mitochondrial dysfunction and neuroinflammation to underpin the neurodegeneration [17]. Here we report that CS-GAG digestion within the nigrostriatal tract reduced SNc cell death and striatal dopaminergic terminal loss in mice bearing a partial, but not a full 6-OHDA lesion. This study shows for the first time that ChABC positively affects cell survival in a more translational animal model of PD.

The lack of protective efficacy of ChABC in mice bearing a full 6-OHDA lesion most likely reflects the severity and speed of the developing lesion. Although ChABC-mediated digestion of the CSPGs occurs rapidly (within 24 h; data not shown), it is clear that any resultant ChABC-mediated plasticity cannot counter the effects of a full lesion. This lack of efficacy is consistent with a previous study that showed no beneficial effect of ChABC treatment alone or in combination with DA grafts in a full 6-OHDA lesion model of PD [9]. Although only partial CSPG digestion had been achieved in this previous study, complete CSPG digestion, as achieved here, was clearly still not sufficient to afford protection in a full 6-OHDA lesion model.

In contrast, when administered acutely at the time of 6-OHDA infusion, ChABC did offer protection in the partial lesion model. Specifically, protection was afforded against the TH-positive cell loss in the rostral SNc, but not in either the medial or caudal levels of the SNc. Given that the pilot study revealed complete CS-GAG digestion throughout the extent of the nigrostriatal axis 35 days after ChABC injection, this regionally-restricted action of ChABC was unexpected. However, as one of the two ChABC injection sites was positioned near to the rostral SNc it is possible that these cell bodies were exposed to the enzyme more readily and to a higher degree than the rest of the SNc, providing these with optimal protection. Interestingly, the accompanying preservation of TH-positive fibres afforded by ChABC treatment was also restricted to the rostral striatum. Whether this is also a consequence of the location of ChABC injection or a reflection of the topography of dopaminergic projections from the SNc to striatum requires further investigation.

The molecular mechanisms behind ChABC’s protective effect have not been explored here. However, digestion of the CS-GAGs is believed to liberate ligands such as various growth factors that aid plasticity [23–25]. Given that several growth factors are known to enhance survival of dopaminergic neurones, such as glial-derived neurotrophic factor, cerebral dopaminergic neurotrophic factor, platelet-derived growth factor-BB and fibroblast growth factor 20 [26–30], the liberation of these growth factors following digestion could certainly explain the protective effects seen here. Further studies will be required to establish the underlying mechanisms. Importantly, ChABC treatment is not anticipated to elicit any aberrant sprouting in the intact nigrostriatal system, but to induce plasticity solely in the lesioned tract. This rationale is based upon the work of Barrit et al. [31] who found that while ChABC caused sprouting of corticostriatal tracts in animals bearing a spinal cord injury, it did not cause sprouting in any system in control, uninjured animals.

Despite an increase in numbers of surviving dopaminergic neurones in the rostral nigrostriatal tract, ChABC treatment did not result in any behavioural improvement in either the amphetamine-induced rotation or cylinder test assessments. This lack of motor improvement may reflect the modest sparing afforded by ChABC treatment of both rostral SNc cells (reaching only 52% of the intact hemisphere cell number) and striatal TH fibres (reaching only 36% of the intact hemisphere equivalent). Conversely this may reflect the fact that there was still a relatively large lesion size in the partial lesion model used here, with only 25% cells in the SNc and 36% striatal TH fibres remaining in the saline-treated animals bearing a partial lesion. Indeed, recent neuroprotective studies utilising the unilateral 6-OHDA mouse model which identified a significant improvement in behavioural phenotype following various treatments support the starting lesion size being the main obstacle to achieving a behavioural improvement. In these previous studies, the lesions were less severe to start with (> 55% SNc cells and > 45% TH-positive fibres remaining in intact hemispheres of the control animals) and so, with an even more modest degree of protection than observed here (between 22 and 26% increase in SNc cells remaining in treated animals and between 20 and 35% sparing of TH-positive fibres), this was sufficient to elevate nigrostriatal tract functionality [32–34]. It will therefore be important for subsequent studies to analyse the effect of ChABC neuroprotection in animals possessing an even milder lesion than utilised here in order to establish whether ChABC has the potential to improve behavioural outcomes.

A number of alternative avenues for improving the behavioural outcome with ChABC treatment deserve consideration. Firstly, as the ChABC enzyme is believed to become denatured quickly under physiological conditions, increasing the exposure to ChABC through varied means which have proven successful against spinal cord injury such as repeated intrathecal administration [35], gene therapy [36–38] or trehalose-thermostabilising [39], may be beneficial. However, given that we had intense C4S immunoreactivity up to 4-weeks after an acute bolus ChABC delivery in the brains of both our full and partial 6-OHDA lesion mice, we are confident that enhanced ChABC exposure is not likely to be required. Indeed, it is quite possible that the expression of CSPGs and their rate of de novo synthesis following digestion is different within the brain compared to the spinal cord, the latter of which has been reported to be 2 weeks [7]. That said, since our C4S analysis only highlights the degree of digested CS-GAGs remaining and not of newly formed CSPGs, increasing ChABC exposure to counter the inhibitory effects of any newly formed late-stage CSPGs may improve cell and fibre preservation within subsequent studies. A second approach to increase ChABC exposure would be to pre-treat the nigrostriatal pathway prior to lesioning. Introducing a priming phase may help liberate any growth factors before exposure to the toxic insult and consequently aid protection during the initial week of cell and fibre loss.

Finally, given that new neuronal connections are theorised to be of use only once the animal has learnt to use them through successful rehabilitation, future studies exploring the benefits of combining ChABC treatment with rehabilitation are warranted [40, 41]. Utilising a rehabilitation regime may be of great use in this partial lesion model as it has been in spinal cord injury models [6, 42], especially given the fact that frequent exercise alone has been reported to increase growth factor release, reduce SNc cell loss and oxidative stress in 6-OHDA lesion models [43–45].

While the 6-OHDA model utilised here is considerably better in terms of construct validity that the previously explored transection models [12–15], it is nevertheless an acute model of PD that lacks some key chronic pathological features. Future studies investigating whether the efficacy of ChABC is retained in models that incorporate chronic features such as alpha synuclein pathology alongside associated impaired axonal transport will help strengthen support for the translational potential of this therapeutic approach.

## Conclusion

This study has revealed the efficacy of ChABC treatment in the 6-OHDA partial lesion mouse model of PD, whereby digestion of CSPGs has reduced the degree of pathology within the nigrostriatal tract. Although it remains to be established whether combined interventions such as rehabilitation can improve motor outcomes, these findings present promise for ChABC treatment as a potential new avenue for providing neuroprotection in PD. In this respect, it is reassuring that alternative formulations with enhanced stability [46] and alternative longer-term means of ChABC delivery, such as doxycycline-inducible gene delivery [47], are being investigated to achieve a more clinically translatable treatment.

## Methods

### Animals

27 and 34 eight-week-old male C57Bl/6 mice (Charles River, UK) were used within the full lesion and partial lesion studies, respectively. All 61 mice were maintained on a 12:12 h light/dark cycle (07:00 am lights on) with food and water ad libitum. Room temperature and humidity were kept at 22 ± 2 °C and 55 ± 2% respectively. All in vivo studies were performed in accordance with the UK Animals Scientific Procedures Act (1986) and were approved by King’s College London Animal Welfare and Ethical Review Body. All surgical, behavioural and histological procedures were performed whilst blinded to the experimental groups.

### Unilateral 6-hydroxydopamine (6-OHDA) lesioning and chondroitinase ABC (ChABC) treatment

All surgeries were conducted in a randomised block design to reduce bias between the blinded treatment groups. Fully lesioned animals were assigned to ChABC treatment (n = 13) or saline control (n = 14). Partially lesioned animals were assigned to ChABC treatment (n = 17) or saline control (n = 17). Anaesthesia was induced with a 5% isoflurane/oxygen mixture then maintained at 3% isoflurane/oxygen. Body temperature was monitored and maintained at 37 °C with a homeothermic heating blanket (Harvard Apparatus). The surgical site was sterilised with 0.4% chlorhexidine (Hibiscrub) before a midline incision was made along the scalp. The skull was then cleaned and dried with cotton swabs.

To establish a full unilateral 6-OHDA lesion, fine-bore holes (Ø 0.5 mm) were drilled at coordinates AP: − 3.0 mm and ML: + 1.2 mm (relative to bregma and skull surface). A blunt-ended 30G needle was then inserted supranigrally to DV: − 4.5 mm (relative to bregma and skull surface) before 8 μg 6-OHDA·HBr (Sigma-Aldrich) in 1 μl 0.02% ascorbate/saline was administered unilaterally at a rate of 0.5 μl/min. The needle was left in place for 4 min to allow for toxin diffusion.

To establish a partial unilateral 6-OHDA lesion, fine-bore holes (Ø 0.5 mm) were drilled at coordinates AP: + 0.5 mm and ML: + 2.0 mm (relative to bregma and skull surface). A blunt-ended 30 G needle was then inserted intrastriatally to DV: − 3.5 mm (relative to bregma and skull surface) before 4 μg 6-OHDA·HBr in 1 μl 0.02% ascorbate/saline was administered unilaterally at a rate of 0.5 μl/min. The needle was left in place for 4-min to allow for toxin diffusion.

5-min after injection of 6-OHDA, animals from both studies received two intracerebral injections of either saline or ChABC (10 U/ml in saline; Seikagaku) into the same 6-OHDA injected hemisphere. In order to achieve digestion of CSPGs along the entire nigrostriatal tract, 1 μl ChABC was administered into the SNc (AP − 2.3 mm; ML + 1.0 mm and DV − 4.2 mm; relative to bregma and skull surface) and 1 μl ChABC into the striatum (AP + 0.02 mm; ML + 2.2 mm and DV − 3.5 mm; relative to bregma and skull surface). These coordinates were determined by a previously run pilot study that showed these to provide most effective CSPG digestion along the entire nigrostriatal tract (data not shown).

After the surgery, all animals were administered buprenorphine (Vetergesic; 0.1 mg/kg; s.c.) for analgesia. All animals additionally received 1 ml of warmed Hartmann’s solution (Aqupharm 11; s.c.) twice a day for 1 week to maintain hydration.

### Behavioural assessments

Motor dysfunction was assessed using two behavioural tests. The cylinder test measured the degree of forelimb ability as described previously [48]. Briefly, taking the lesion as day 0, on days − 4, − 1, 3, 10, 17, 24 and 31 for the full lesion study and on days − 3, − 1, 14, 21, 28 and 35 for the partial lesion study, mice were placed individually within 2 litre glass beakers (Ø 12 cm) and their forepaw preference was monitored during exploratory rearing behaviour captured through 5-min video recordings. Touches by the forepaw ipsilateral-to-the-lesion, contralateral-to-the-lesion or by both forepaws simultaneously were counted. An asymmetry score was then calculated to indicate the proportional use of each forepaw: a score of 50% indicated no bias and < 50% denoted impairment of the injured (contralateral) forepaw.

Amphetamine-induced rotations were counted on day 35 (full) or day 37 (partial) post-lesion. Animals were placed within cylindrical arenas (Ø 40 cm) in which the motion-tracking tool Ethovision XT6 was used for recording. A custom optimised calibration file was loaded, which allowed the recording of full 360° rotations about the animal’s midpoint. Ipsiversive and contraversive rotations were then individually measured before calculating net ipsiversive rotations. Following a 20-min habituation period, mice were administered with d-amphetamine hemisulphate (Tocris; 5 mg/kg in saline; i.p.) and rotations were recorded for 90-min thereafter.

### Histological assessment of lesion severity

On day 42 post-lesion (full lesion study) or day 39 post-lesion (partial lesion study) all animals were humanely killed following sodium pentobarbital terminal anaesthesia (200 mg; Sigma; i.p.). Animals were then formalin-perfused and their brains post-fixed in 4% paraformaldehyde/PBS before being embedded in paraffin wax blocks. 7 µm thick coronal sections were cut with a microtome (Thermo Scientific) at three rostrocaudal levels of the SNc (rostral − 2.92 mm, medial − 3.16 mm and caudal − 3.52 mm AP; relative to bregma) and three levels of the striatum (rostral + 1.0 mm, medial + 0.5 mm and caudal − 0.22 mm AP; relative to bregma). Sections were then mounted on Poly-l-lysine coated slides (VWR). Three sections from each of the three levels of the striatum (9 in total) were incubated with rabbit polyclonal anti-tyrosine hydroxylase (TH) primary antibody (AB152, Millipore) overnight before being washed in TBS twice and incubated in biotinylated goat anti-rabbit secondary antibody (BA-1000, VectorLabs) for 2 h at room temperature. Following a further two TBS washes, the sections were incubated with streptavidin-biotinylated horseradish peroxidase conjugate (PK6100, VectorLabs) for 30-min at room temperature. Slides were then immersed in diaminobenzidine tetrachloride for 10-min and mounted.

Cells of the SNc and TH-positive fibres of the striatum, in both the lesioned and intact hemispheres, were imaged with 100× and 50× magnification, respectively (Axioskop, light-field compact microscope). For the purpose of side-to-side comparisons where the intention is to quantify percentage loss between the intact and lesioned hemispheres in a given animal, we adopted manual cell counting [20]. Previous studies have reported no difference in outcomes when comparing stereological analysis with manual cell counting in the 6-OHDA lesion model [21]. ImageJ software was used to count the number of viable (i.e. intact round cells with a clear nucleus and cytoplasm) A9 TH-positive SNc cells and to measure mean grey value (MGV) of striatal TH-positive fibres in the dorsal and ventral striatum. In all cases, the operator was blinded to treatment throughout the analysis. An average of all three analysed levels of the SNc/striatum was produced for both the saline- and ChABC-treated groups. In fully lesioned mice, no differences between levels were noted for either striatum or SNc, so data were pooled across the entire rostrocaudal extent, while for partially lesioned mice the levels were analysed independently. Data are expressed as number of cells (SNc) or fibre density [striatum and rostral SNc (in partial lesion study)] in the lesion side as a percentage of the respective intact side.

### Confirmation of CSPG digestion by chondroitin-4-sulphate stub histology

Immunohistochemistry was used to stain for the C4S-stub antigen that remains following ChABC-mediated digestion. Staining for C4S (mouse monoclonal; 1:500; MPBio #636511) was completed using sections adjacent to the TH-stained sections whilst using the same protocol as previously described for TH [29].

To confirm whether the extent of ChABC-mediated digestion of CS-GAGs along the nigrostriatal pathway in vivo was maximal, the effect of subsequent ChABC exposure ex vivo was assessed. Briefly, 7 µm SNc brain sections from saline- and ChABC-treated mice from both the full and partial lesion studies (n = 3 for each group) were incubated with ChABC (10 U/ml; Seikagaku) or tris-buffered saline for 3 h at 37 °C. C4S-stub immunoreactivity was then stained for, as described above.

### Statistical analysis

All quantitative data are expressed as mean ± standard error of the mean (SEM). Statistical analyses were conducted with GraphPad Prism (version 7) software. Behavioural data were analysed using two-way repeated measures ANOVA, SNc cell count data and TH density were analysed by unpaired Student *t*-tests and striatal TH-positive fibre MGV data were analysed by two-way ANOVA. Post-hoc tests were applied when appropriate as detailed in figure legends.

## Data Availability

All datasets used within this study are available from the corresponding author on reasonable request.

## Abbreviations

6-OHDA: 6-hydroxydopamine
ChABC: chondroitinase ABC
CS-GAGs: chondroitin sulphate glycosaminoglycans
CSPGs: chondroitin sulphate proteoglycans
MGV: mean grey value
PD: Parkinson’s disease
SNc: substantia nigra pars compacta
TH: tyrosine hydroxylase
